# Glucocorticoid-induced leucine zipper regulates liver fibrosis by suppressing CCL2-mediated leukocyte recruitment

**DOI:** 10.1038/s41419-021-03704-w

**Published:** 2021-04-29

**Authors:** Sara Flamini, Philipp Sergeev, Zenobio Viana de Barros, Tommaso Mello, Michele Biagioli, Musetta Paglialunga, Chiara Fiorucci, Tatiana Prikazchikova, Stefano Pagano, Andrea Gagliardi, Carlo Riccardi, Timofei Zatsepin, Graziella Migliorati, Oxana Bereshchenko, Stefano Bruscoli

**Affiliations:** 1 Department of Medicine and Surgery, University of Perugia, Severi Place 1, 06132 Perugia, Italy; 2 Laboratory of Immune Regeneration and Experimental Hematology, Department of Pediatric Hematology and Oncology, Bambino Gesù Children Hospital, Viale San Paolo 15, Roma, 00146 Italy; 3 Skolkovo Institute of Science and Technology, Bolshoy Boulevard 30b1, 121205 Moscow, Russia; 4 Institute for Molecular Medicine Finland, Helsinki Institute of Life Science, University of Helsinki, Helsinki, FI-00014 Finland; 5 Gastroenterology Research Unit, Department of Experimental and Clinical Biochemical Sciences; Center of Excellence for Research, Transfer and High Education, DENOthe, University of Florence, Florence, 50139 Italy; 6 Department of Chemistry, Lomonosov Moscow State University, 119992 Moscow, Russia; 7 Department of Philosophy, Social Sciences and Education, Ermini Place 1, 06123 Perugia, Italy

**Keywords:** Chronic inflammation, Experimental models of disease

## Abstract

Liver fibrosis (LF) is a dangerous clinical condition with no available treatment. Inflammation plays a critical role in LF progression. Glucocorticoid-induced leucine zipper (GILZ, encoded in mice by the *Tsc22d3* gene) mimics many of the anti-inflammatory effects of glucocorticoids, but its role in LF has not been directly addressed. Here, we found that GILZ deficiency in mice was associated with elevated CCL2 production and pro-inflammatory leukocyte infiltration at the early LF stage, resulting in enhanced LF development. RNA interference-mediated in vivo silencing of the CCL2 receptor CCR2 abolished the increased leukocyte recruitment and the associated hepatic stellate cell activation in the livers of GILZ knockout mice. To highlight the clinical relevance of these findings, we found that *TSC22D3* mRNA expression was significantly downregulated and was inversely correlated with that of *CCL2* in the liver samples of patients with LF. Altogether, these data demonstrate a protective role of GILZ in LF and uncover the mechanism, which can be targeted therapeutically. Therefore, modulating GILZ expression and its downstream targets represents a novel avenue for pharmacological intervention for treating LF and possibly other liver inflammatory disorders.

## Introduction

Liver fibrosis (LF) is characterized by excessive scar tissue formation during liver repair. LF is associated with most chronic liver diseases, including viral infections, alcoholic liver cirrhosis, nonalcoholic fat liver disease (NAFLD), and autoimmune hepatitis. Although asymptomatic, LF represents a dangerous medical condition leading to irreversible and potentially lethal liver cirrhosis^1,2^. Understanding the molecular regulatory mechanisms in the fibrotic processes is crucial for developing effective preventive and therapeutic strategies.

A complex interplay among different cell types regulates LF. The replacement of hepatocytes damaged by hepatotoxic agents, e.g., hepatitis viruses, alcohol metabolites, bile acids, and some pharmacologic agents, activates an inflammatory response and white blood cell recruitment in the earliest stages following liver injury^3^. Inflammation plays an important role in the development of virtually all forms of fibrosis^4^. Both innate and adaptive immunity cells are found in lesions at the early stages of LF development, and include monocytes/macrophages, natural killer/natural killer T (NK/NKt) cells, and T and B lymphocytes^3^. Infiltrating immune cells produce pro-inflammatory and pro-fibrotic cytokines and chemokines, leading to the activation of hepatic stellate cells (HSCs), fibroblasts, and myofibroblasts, which proliferate and produce large amounts of extracellular matrix components^3^. The fine balance between specific cytokines produced by resident and infiltrating macrophages and T and NK cells defines the resolution or enhancement of inflammatory and fibrotic processes. Cytokines that promote fibrosis include TGF-β1, IL-6, TNF-α, and IL-1, produced by activated macrophages, and IL-4 and IL-13, associated with the T helper type 2 (Th2) response^1,5,6^. While it is not possible to clearly distinguish between the relative contribution of the innate and adaptive immune systems to inflammatory response propagation in LF development, identifying the factors regulating leukocyte recruitment to the liver provides insight into the mechanisms that restrain LF.

Chemokines regulate circulating immune cell migration and activity during fibrosis development. Monocyte chemoattractant proteins (MCPs) are potent chemoattractants of monocytes, activated T cells, NK cells, and immature dendritic cells^7^. MCP-1 (or CCL2) binds to its receptor (CCR2); besides recruiting leukocytes, it also directly influences fibrogenic HSC activity. Experimental models using CCL2- or CCR2-deficient mice have demonstrated the functional relevance of the CCL2–CCR2 pathway in LF^3,7–11^. Therefore, strategies that dampen excessive inflammatory responses and immune cell recruitment to the liver represent therapeutic potential for LF management.

Glucocorticoids (GCs) are potent anti-inflammatory drugs used for treating a wide spectrum of inflammatory diseases^12–14^, including those of the liver, e.g., severe alcoholic hepatitis^15^, autoimmune hepatitis^16^, and liver failure^17,18^. Their efficacy in treating LF is attributed to their ability to inhibit leukocyte migration to inflammation sites and suppress pro-inflammatory mediator production. Treating mice with the synthetic GC prednisolone suppressed inflammatory responses in a model of carbon tetrachloride (CCl_4_)-induced hepatitis, but surprisingly exacerbated liver injury and delayed liver repair^19^. GCs have a controversial effect on LF because they exert opposing effects on immune cells and on HSCs^20^. Overall, the serious adverse effects of GC therapy represent the limiting factor to their prolonged usage for treating liver inflammation. Therefore, there is a strong need to identify means of disassociating the anti-inflammatory and metabolic effects of classical GCs to improve the therapy of liver diseases, including LF.

Glucocorticoid-induced leucine zipper (*Gilz* or *Tsc22d3*) is a gene rapidly induced by GCs in T cells^21,22^. GILZ functions in immune^21,22^ and nonimmune cells, including germ^23^, muscle^24–26^, and endothelial cells^27^ and adipocytes^28^. GILZ mimics the effects of GCs in many cellular processes regulating apoptosis^29,30^, and cell activation and differentiation^21,31–39^. These effects are partly dependent on the ability of GILZ to repress the ERK–MAPK–NF-κB pathways important for leukocyte activation, migration, and pro-inflammatory cytokine and chemokine expression^40–43^. The immunomodulatory effects of GILZ have been demonstrated in in vivo models of inflammation^35,44^.

The role of GILZ in LF development has not been directly investigated. Deregulated GILZ expression was evidenced in autoimmune hepatitis patients^45^, and the GC receptor–GILZ pathway was implicated in regulating liver inflammation in obese mice^46^. We addressed the role of GILZ in LF development in a mouse model of CCl_4_-induced LF^23^. We found that GILZ deficiency in *Tsc22d3* knockout (hereafter: GILZ KO) mice is associated with more pronounced LF, characterized by increased CCL2 expression and leukocyte liver infiltration. Downregulation of CCR2 expression via RNA interference (RNAi)-mediated silencing in vivo reverted the enhanced leukocyte recruitment in GILZ KO mice and the associated HSC activation. Moreover, human gene expression data analysis revealed that *TSC22D3* mRNA expression is downregulated in NAFLD patients with LF and correlates inversely with that of *CCL2*. Therefore, our data show that GILZ restrains LF development by controlling CCL2-dependent leukocyte trafficking into the liver, and that this pathway can be targeted therapeutically to restrain LF development and/or progression.

## Materials and methods

### Mice

Mice were kept in Center for Preclinical Studies of the University of Perugia and treated according to the Italian (D.M. 116.192), European (Official Journal of European Community 358/1, December 18, 1986), and American (Animal Welfare Assurance No. A5594-01, Department of Health and Human Services, Washington, DC) laws, approved by University of Perugia. Male C57BL/6 GILZ KO and wild-type (WT) littermate mice were generated, as previously described^23^. Mice overexpressing GILZ (GILZ transgenic, GILZ TG) were previously described^47^.

### Liver fibrosis induction

LF was induced by CCl_4_ administration (FLUKA, Cod. No. 87030) according to a previously published method^48^. For this purpose, the mice were administered i.p. 500 μL/Kg body weight of CCl_4_ in an equal volume of olive oil.

### Tissue histology

Portions of the right and left liver lobes were fixed in 10% formalin, embedded in paraffin, sectioned, and stained with Sirius red for morphometric analysis.

### Immunohistochemistry

Paraffin-embedded tissue sections 7 μm thick were rehydrated, boiled in Tris/EDTA pH = 9 for 20 min, incubated in 3% H_2_O_2_ for 10 min to inhibit endogenous peroxidases, and then incubated with a blocking solution (2.5% normal goat serum, Vector Laboratories). Monoclonal anti-α-SMA (clone 1A4, cat. A2547 Sigma-Aldrich, dil. 1:200) was incubated overnight at 4° C in blocking solution. The secondary antibody anti-Mouse IgG ImmPRESS Reagent Kit (Vector Laboratories, cat. MP7402) was incubated for 30 s at RT and the chromogenic reaction developed with DAB (Sigma-SIGMAFAST™ DAB). Slides were then counter-colored with hematoxylin (Gill’s 3, Bio-Optica), dehydrated, and mounted in DPX.

Frozen tissue was sectioned (7 μm thick), air dried, and fixed in cold acetone (−20° C) for 20 min. Slides were then air dried and kept in 0.3% H_2_O_2_ in methanol for 30 min to inhibit endogenous peroxidases. Following 2.5% NGS blocking, anti-α-SMA was incubated overnight at 4° C at 1:500 dilution. The secondary antibody anti-mouse IgG ImmPRESS Reagent Kit (Vector Laboratories, cat.MP7402) was incubated for 30 s at RT and the chromogenic reaction developed with DAB (Sigma-SIGMAFAST™ DAB with metal Enhancer). Section was counterstained with Nuclear Fast Red (Vector Laboratories), dehydrated, and mounted in DPX (cat. 06522, Sigma -Aldrich).

### RNA isolation and qPCR

RNA was isolated from whole tissue fragment using RNA-XPress™ Reagent (MB601, HIMEDIA) and reverse-transcribed using PrimeScript RT reagent Kit, with gDNA Eraser (Perfect Real-Time-TAKARA). Quantitative real-time PCR (qPCR) was performed using the 7300 Real-Time PCR System (Applied Biosystems), SYBR™ Select Master Mix (Applied Biosystems), and TaqMan™ Gene Expression Master Mix(Applied Biosystems). The qPCR TaqMan probes (Applied Biosystems) were as follows: Fas, Mm01204974; FasL, Mm00438864; Bcl-xL, Mm00437783; and Actb, 4352341E. Primers using in amplification using Sybergreen methods are listed in Supplementary Table 2.

### Isolation of liver infiltrating leukocytes

The harvested livers were digested by liver dissociation kit (Miltenyi Biotec), following manufacturer’s instruction. Briefly, homogenized livers were incubated with dissociation mix for 45 min, the cell suspensions were applied to a 100 μm and then 70 μm cell strainer. Released cells were layered on a 40–80% Percoll gradient and spun at 400 × *g* for 20 min to get an enriched LPMC population and processed as described^49^.

### Isolation of liver cells

Primary hepatocytes and Kupffer cells were isolated via a two-step collagenase liver perfusion. Briefly, mice were anesthetized with ketamine/xylazine, and perfused via the portal vein first with Liver Perfusion Medium (Gibco cat. 17701038) to remove the blood and then with a collagenase solution (cat. C5138 Sigma-Aldrich, 0.5% w/v in minimum essential medium). The cell suspension was filtered through a 70 μm cell strainer (Greiner Bio-one) and centrifuged to separate hepatocytes (pellet) from non-parenchymal cells (supernatant). Pelleted hepatocytes were purified through centrifugation over 40% Percoll and plated in a collagen sandwich, while Kupffer cells were purified from the non-parenchymal cells pool by differential adhesion on plastic. RNA from purified hepatocytes and KC was extracted 24 h after plating.

### Antibodies and flow cytometry

Cells were prepared and stained with directly conjugated antibodies as described^50^. Monoclonal antibodies were purchased from Thermo Fisher: anti-CD3e (145-2C11), anti-CD4 (GK1.5), anti-CD8 (53-6.7), anti-CD11b (M1/70), anti-B220 (RA3-6B2), anti-CD49b (DX5), and anti-Gr1 (RB6-8C5). Analyses were performed using the ATTUNE NxT three-laser standard configuration (Life Technologies), and data were analyzed using Flow Jo software (Tree Star).

### siRNA description and LNP formulation

We used one small interfering RNA (siRNA) described by Leuschner et al.^51^ and in Supplementary Methods. siRNA was selected to avoid off-target activity based on several known criteria as described previously^52^. siRNA modification with 2′-OMe pyrimidine nucleotides and 3′-internucleotide phosphorothioates should reduce immune response and increase stability in vivo. Potency of siRNA targeting CCR2 were studied by transfection in Hepa1–6 cells followed by qPCR analysis after 24 h. siRNA were formulated in lipid nanoparticles (LNPs), as previously described^52^. Particle size measurements were performed using a Zetasizer Nano ZSP (Malvern Panalytical) according to the manufacturer’s protocol, siRNA entrapment efficiency was determined using the Quant-iT™ RiboGreen® reagent (R11491, Thermo Fisher Scientific) as described^52^.

### Analysis of gene expression using available gene expression data

Transcriptional profile of NASH patients was downloaded from Gene Expression Omnibus (GEO) with accession number GSE48452. We analyzed total RNAseq data from 64 human liver samples grouped into “control” (healthy individuals; *n* = 14), “healthy obese” (*n* = 27), “NASH—high fat score” (patients presenting NASH and with a fat score equal to or higher than 70; *n* = 9), and “NASH—fibrosis 1–4” (patients with a fibrosis score ranging from 1 to 4; *n* = 14). Transcriptional profile of NAFLD patients was downloaded from the GEO with accession number GSE49541. This dataset represents two clinically defined pathological groups at the extremes of NAFLD: mild NAFLD (fibrosis stages 0–1; *n* = 40), with little risk of developing severe liver disease; advanced NAFLD (fibrosis stages 3–4; *n* = 32), with significant likelihood of developing liver-related morbidity and mortality. Dataset passed through Kolmogorov–Smirnov test, and statistical analysis was performed by parametric ANOVA test for multiple comparisons and or Mann–Whitney test using GraphPad Prism 6.0 software. Pearson’s correlation coefficient for gene expression correlation (*R*) and Tukey’s multiple comparison test were performed in GraphPad Prism 6.0.

### Statistical analysis

Statistical analysis was performed with Prism 6.0 (GraphPad). The two-tailed unpaired Student *t* test or nonparametric Mann–Whitney *U* test was used for statistical comparisons. For multiple comparisons, two-way ANOVA was performed.

## Results

### GILZ deficiency is associated with enhanced LF development in mice

**Fig. 1:**
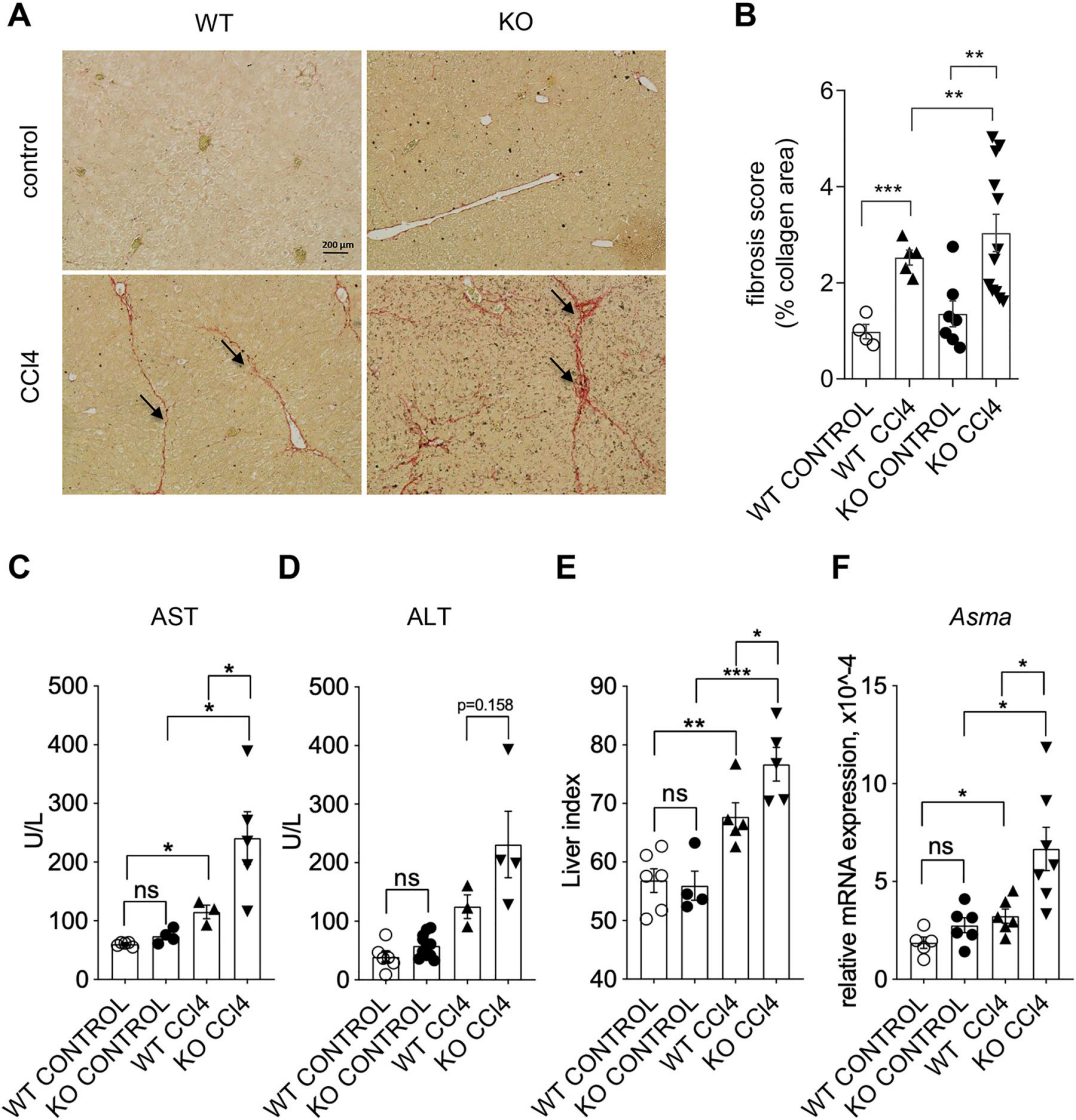
Lack of GILZ results in increased CCl_4_-induced liver fibrosis development. **A** Sirius Red staining of liver sections from 4-month-old WT and GILZ KO mice that had been treated with oil (control; upper panels) or CCl_4_ (lower panels) for 6–7 weeks. Arrows indicate the areas of collagen deposition. **B** Graph shows the quantitative measurement of collagen deposition in all experimental groups. **C**, **D** AST (**C**) and ALT (**D**) levels in blood from 4-month-old WT and GILZ KO mice that had been treated with CCl_4_ for 6–7 weeks. **E** LI of 4-month-old WT and GILZ KO mice that had been treated with oil (control) or CCl4 for 6–7 weeks. **F** qPCR analysis of *Asma* mRNA expression in livers from 4-month-old WT and GILZ KO mice that had been treated for 6–7 weeks with oil (control) or CCl_4_. All data are presented relative to *Actb* mRNA expression. Each dot represents an individual mouse; bars indicate the mean. Results are presented as the means ± SEM of three (**A**, **B**, **F**) or two (**C**–**E**) independent experiments (*p < 0.05, ***p < 0.001, ns not significant).

GILZ is expressed both in hepatocytes and in Kuppfer cells (Fig. S1). To evaluate possible contribution of GILZ in the development of LF, WT, and GILZ KO mice were treated with CCl_4_ for 6–7 weeks to induce LF as described^48^. The degree of LF development upon CCl_4_-induced chronic liver injury was examined evaluating the degree of collagen deposition in the livers of WT and GILZ KO mice using Sirius Red staining. We found that the GILZ KO mice showed increased collagen deposition in the livers, following CCl_4_ treatment compared to similarly treated WT mice (Fig. 1A, B). To investigate whether the absence of GILZ is associated with compromised liver function following CCl_4_-incurred damage, serum levels of markers of liver damage, aspartate aminotransferase (AST), and alanine aminotransferase (ALT)^53^, were evaluated in WT and GILZ KO mice treated for 6–7 weeks with CCl_4_. The AST serum levels were significantly increased in GILZ KO mice upon CCl_4_ treatment compared to similarly treated WT mice (Fig. 1C), while the ALT serum levels showed a tendency increase in GILZ KO mice compared to similarly treated WT mice (Fig. 1D). In addition, Liver Index (LI), representing the ratio of liver weight and body weight × 1000 (ref. ^54^), was also significantly increased in GILZ KO mice following CCl_4_ treatment compared to similarly treated WT mice (Fig. 1E). Since the increase in α-smooth muscle antigen (α-SMA) expression characterizes active fibrosis process, we compared the level of mRNA expression of *Asma* in livers isolated from WT and GILZ KO mice treated with CCl_4_ for 7 weeks. As expected, *Asma* mRNA expression was significantly increased in both WT and GILZ KO mice following CCl_4_ treatment; however, *Asma* levels were significantly higher in GILZ KO compared to similarly treated WT mice (Fig. 1F). Taken together, these data demonstrate that the absence of GILZ results in an enhanced development of experimentally induced LF in mice, and suggest that GILZ plays a role in LF development process.

### GILZ deficiency is associated with elevated HSC activation

**Fig. 2:**
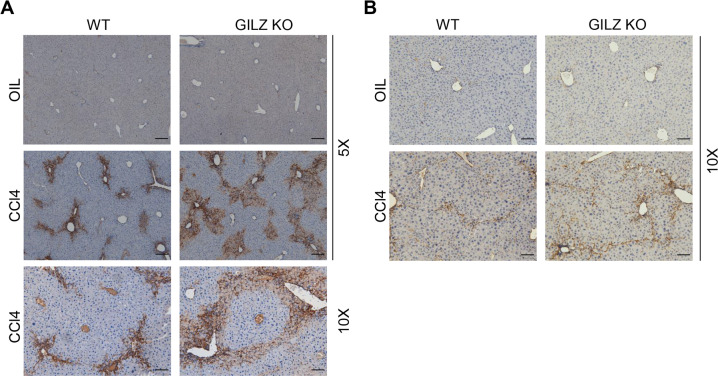
GILZ deficiency is associated with elevated HSC activation. **A**, **B** Immunohistochemical analysis of α-SMA expression in the liver sections of WT and GILZ KO mice that had been treated with oil or CCl_4_ for 72 h (**A**) or 7 weeks (**B**). Original magnification, 5× or 10×, as indicated. Arrows indicate the areas of α-SMA positivity. Scale bar, 100 μm (5× magnification images) or 200 μm (10× magnification images).

HSC play a key role in the fibrotic process following liver injury^55,56^. α-SMA expression serves as a marker of HSCs in normal and pathologic liver tissue. To demonstrate that enhanced CCl_4_-induced LF development observed in GILZ KO mice is associated with HSC activation, we performed immunohistochemistry analysis of α-SMA expression in livers isolated from WT and GILZ KO mice treated with oil (control) or CCl_4_ (Fig. 2). The data show that the absence of GILZ is associated with increased expression of α-SMA and HSC expansion in the pericentral zone of the liver lobule, consistent with CCL_4_-induced damage pattern, both at acute (Fig. 2A) and chronic (Fig. 2B) phases of LF development. These results show that the absence of GILZ is associated with elevated expression of α-SMA during LF development, suggesting that GILZ restrains HSC activation.

### GILZ deficiency results in increased leukocytes recruitment in liver upon CCl_4_ treatment

To investigate the mechanisms underlying the enhanced LF development in GILZ KO mice, we first examined whether the absence of GILZ is associated with enhanced susceptibility to CCl_4_-induced liver injury and cell death. Histological analysis of tissue sections did not reveal morphological differences in livers of WT and GILZ KO mice treated with oil or CCl_4_ (data not shown). Moreover, we compared the expression of several apoptosis-related genes in livers of control or CCl_4_-treated WT and GILZ KO mice. qPCR analysis revealed that mRNA expression levels of *Fas* (Fig. S2A), *Fasl* (Fig. S2B) and *Bcl2l1* (Fig. S2C) did not significantly differ in livers of WT and GILZ KO mice. Thus, we did not find evidence of differences in initial CCl_4_-induced cell death between WT and GILZ KO mice.

**Fig. 3:**
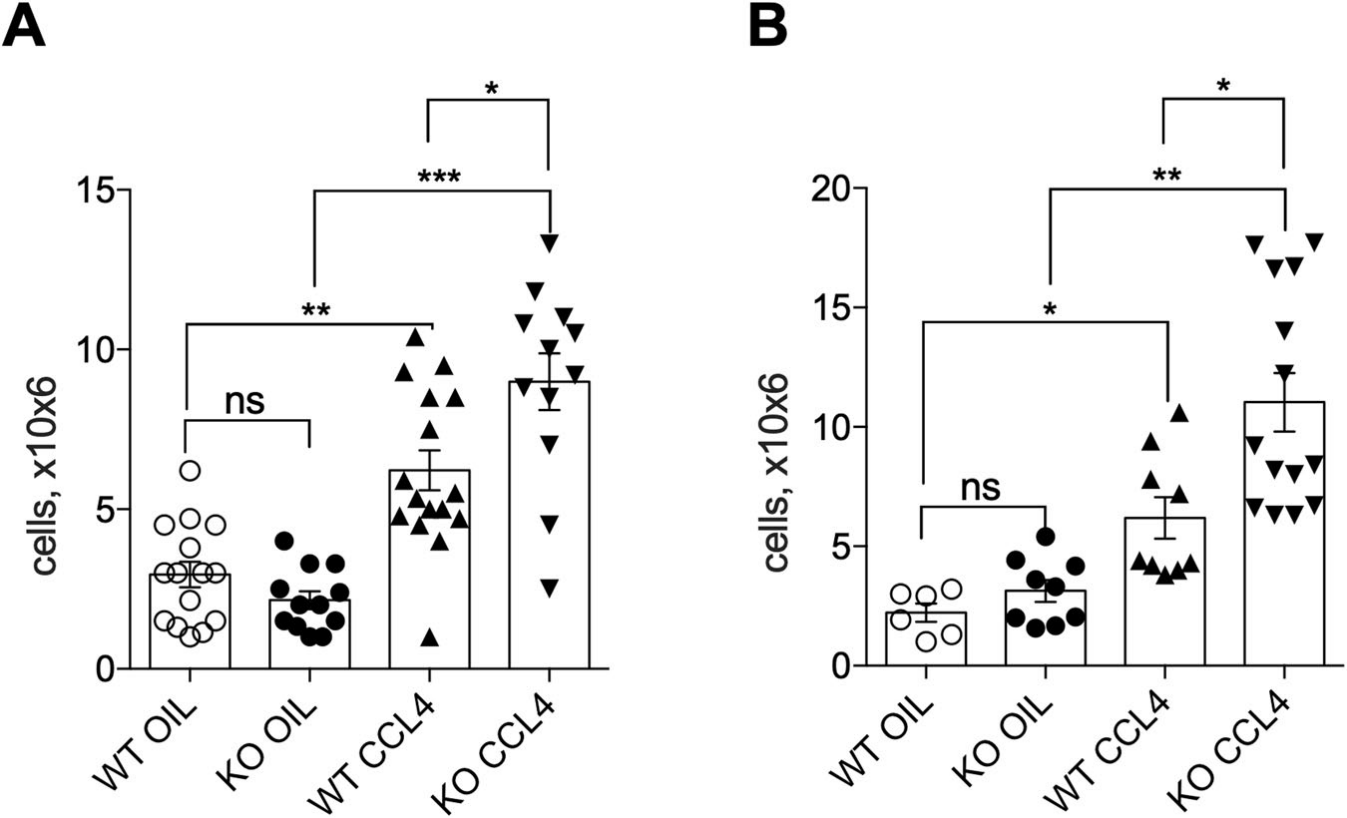
Lack of GILZ results in increased leukocyte infiltration in the liver upon CCl_4_ treatment. **A**, **B** Number of infiltrated leukocytes in livers from 4-month-old WT and GILZ KO mice that had been treated with oil (control) or CCl_4_ for 72 h (**A**) and 6–7 weeks (**B**). Results are presented as the means ± SEM. Each dot represents an individual mouse; bars indicate the mean. Data were pooled from five (**A**) or three (**B**) independent experiments (*p < 0.05, **p < 0.005, ***p < 0.001, ****p < 0.0001, ns not significant).

Since leukocytes play a role in the initiation of inflammatory processes, HSC activation and LF progression^57^, and since GILZ has been implicated in the regulation of leukocytes trafficking^34^, we hypothesized that GILZ regulates leukocytes recruitment to liver at the initial stages of LF development. We evaluated the number of leukocytes in livers of WT and GILZ KO mice 72 h after treatment with oil or CCl_4_. As expected, CCl_4_ treatment led to a significant increase in the number of leukocytes in livers of WT mice, as compared to control mice (Fig. 3A). This initial CCl_4_-induced recruitment was significantly enhanced in GILZ KO mice (Fig. 3A). The increased leukocyte presence in liver persisted also long term in GILZ KO mice treated with CCl_4_ for 6–7 weeks, as compared to similarly treated WT mice (Fig. 3B). These results indicate that the lack of GILZ is associated with enhanced leukocyte presence in the liver following CCl_4_-induced liver damage, and suggest that GILZ contributes to the regulation of the inflammatory process during LF development.

### Lack of GILZ results in increased recruitment of monocytes and CD4 + T cells to liver

**Fig. 4:**
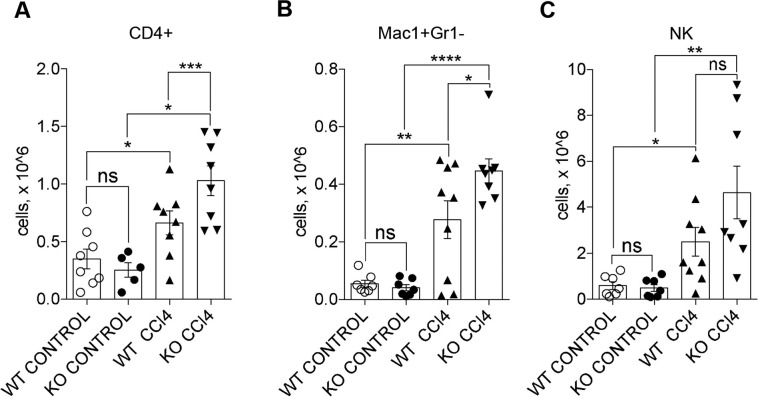
Lack of GILZ results in increased monocyte, CD4^+^ T cell, and NK cell recruitment to the liver. **A**–**C** Number of CD4^+^ (**A**), Mac1^+^Gr1^−^ (**B**), and NK (**C**) cells infiltrated in the livers of 4-month-old WT and GILZ KO mice that had been treated with oil (control) or CCl_4_ for 72 h. Data were pooled from three independent experiments. Results are presented as the means ± SEM; each dot represents an individual mouse; scale bars indicate the mean (*p < 0.05, **p < 0.005 ***p < 0.001, ****p < 0.0001, ns not significant).

In order to identify the immune cell subpopulations recruited to liver upon CCl_4_ treatment in WT and GILZ KO mice, we have performed flow cytometry analysis to evaluate the number of liver infiltrating monocytes (Mac1^+^Gr1^−^), granulocytes (Mac1^+^Gr1^+^), NK (CD49d^+^), NKt (CD49d^+^CD3e^+^), and T cells (CD3e^+^; Fig. S3). Number of CD3^+^ T cells in liver showed a tendency increase in GILZ KO mice compared to similarly treated WT mice (Fig. S3A). However, if CD3^+^ T cells were further subdivided into CD4^+^ and CD8^+^ cells, we detected a significant increase in the number of CD4^+^ cells in livers derived from CCl_4_-treated GILZ KO mice compared to those derived from similarly treated WT mice (Fig. 4A). Instead, the number of CD8^+^ cells did not significantly differ between WT and GILZ KO livers (Fig. S4B). Since Mac1^+^ cells represent the predominant subset in early hepatic lesions^58^, we compared the number of granulocytes (Mac1^+^Gr1^+^) and monocytes/macrophages (Mac1^+^Gr1^−^) present in the liver upon 72 h CCl_4_ treatment. The number of Mac1^+^Gr1^+^ cells did not significantly differ between WT and GILZ KO mice (Fig. S4C). Instead, the number of Mac1^+^Gr1^−^ cells was significantly increased in livers of CCl_4_-treated GILZ KO mice compared to similarly treated WT mice (Fig. 4B). Moreover, GILZ deletion also mildly affected the number of NK cells in the livers of CCl_4_-treated mice, which revealed tendency increase in GILZ KO compared to similarly treated WT mice (Fig. 4C). NKt cells did not differ in their ability to infiltrate liver in WT and GILZ KO mice upon CCl_4_ treatment (Fig. S4D). Interestingly, the increase in CD4^+^ cells in the livers of GILZ KO mice was associated with a significant increase in the mRNA expression of IL-4 cytokine in both early and late stages of LF development (Fig. S5A, B), and a modest decrease in IFN-γ mRNA expression at the early stage (Fig. S5C), suggesting a predominance of the Th2-type response in the livers of mice lacking GILZ. To confirm that GILZ regulates recruitment of these leukocyte subpopulations to liver, we have used GILZ TG mice^47^. These mice presented a fourfold increase in GILZ protein levels in livers (Fig. S6A, B), which was sufficient to significantly suppress liver infiltration of T, NK cells, and macrophages in CCl_4_-treated GILZ TG mice compared to similarly treated WT mice (Fig. S6C–E). Altogether these results indicate that GILZ regulates the recruitment of monocytes, T and NK cells to livers challenged with CCl_4_.

### Lack of GILZ results in increased CCL2 expression

**Fig. 5:**
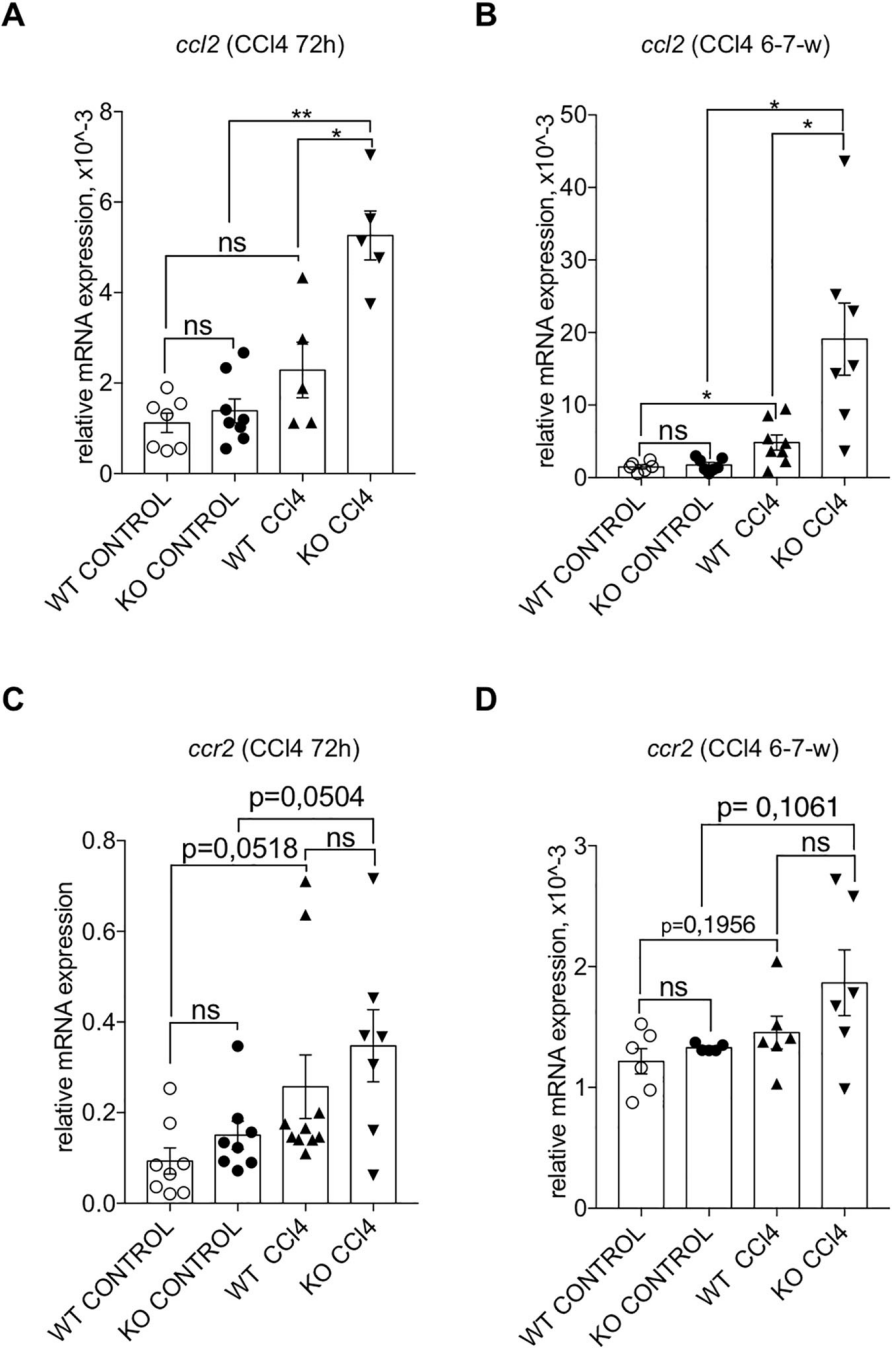
Lack of GILZ results in increased *Ccl2* mRNA expression in the liver following CCl_4_ treatment. **A**, **B** qPCR analysis of *Ccl2* mRNA expression in the livers of 4-month-old WT and GILZ KO mice that had been treated for 72 h (**A**) or 6–7 weeks (**B**) with oil (control) or CCl_4_. **C**, **D** qPCR analysis of *Ccr2* mRNA expression in the livers of 4-month-old WT and GILZ KO mice that had been treated for 72 h (**C**) or 6–7 weeks (**D**) with oil (control) or CCl_4_. All data are presented relative to *Actb* mRNA expression. Each dot represents an individual mouse; scale bars indicate the mean. Results are presented as the means ± SEM. Data were pooled from three (**A**–**C**) or two (**B**–**D**) independent experiments (*p < 0.05, **p < 0.005, ns not significant).

Since liver injury leads to an upregulated expression of various chemokines^59^, and since GILZ controls the expression of several pro-inflammatory mediators, including chemokines^22^, we checked the mRNA expression levels of different chemokines and their receptors involved in fibrotic progression in the livers of WT and GILZ KO mice following CCl_4_ treatment. No significant differences in mRNA expression of *Cxcl12* (Fig. S7A), *Ccl5* (Fig. S7B), and its receptor *Ccr5* (Fig. S7C) were found in livers of similarly treated WT and GILZ KO mice. qPCR analysis has revealed that *Ccl2* mRNA expression was significantly increased in livers of WT mice upon CCl_4_ treatment (Fig. 5A), confirming that its expression is modulated upon LF induction. Interestingly, GILZ KO mice exhibited a significantly higher induction of *Ccl2* mRNA expression compared to similarly treated WT mice (Fig. 5A). The mRNA expression levels of CCL2 receptor CCR2 did not significantly differ in livers of WT and GILZ KO mice, both at steady state and upon CCl_4_ treatment (Fig. 5B). These results demonstrate that GILZ controls the expression of CCL2 chemokine during hepatic injury. Consistent with previous experimental evidences^22^, we detected a tendency increase in the levels of phosphorylated ERK in the livers of CCl_4_-treated GILZ KO mice, compared to similarly treated WT mice, suggesting that deregulation of MAPK/ERK pathway might be related to the activation of CCl2 expression in the absence of GILZ (Fig. S8A, B).

### CCR2 inhibition reverts enhanced CCl_4_-induced leukocytes infiltration into the liver in GILZ KO mice

**Fig. 6:**
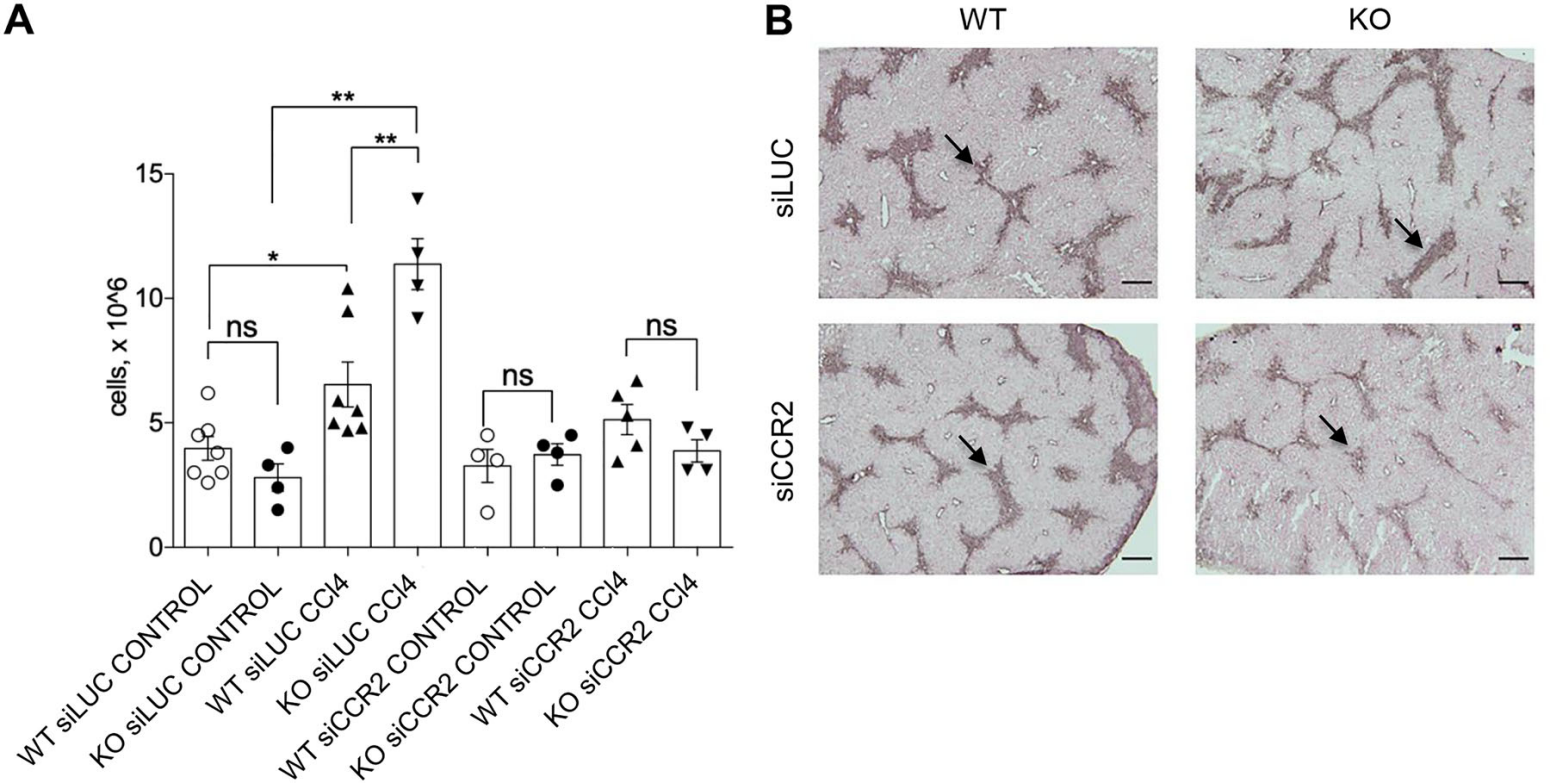
Silencing CCR2 reverts enhanced leukocyte infiltration in GILZ KO mouse livers. **A** The number of leukocytes isolated from the livers of 4-month-old WT and GILZ KO mice that had been pretreated with siCCR2 or siLUC (LUC) for 1 week and then treated with oil (control) or CCl_4_ for 72 h. **B** Immunohistochemical analysis of α-SMA expression in the liver sections of WT and GILZ KO mice that had been pretreated with siLUC or siCCR2, and then treated with oil or CCl_4_ for 72 h. Arrows indicate the areas of α-SMA positivity. Original magnification, 5×; scale bar, 200 µM. Results are presented as the means ± SEM. Each dot represents an individual mouse; scale bars indicate the mean. Data were pooled from two independent experiments (**p < 0.005, ns not significant).

In order to demonstrate that the augmented recruitment of CD4^+^ T cells and Mac1^+^ cells observed in GILZ KO mice depends on elevated CCL2 expression, we performed in vivo silencing of the CCL2 receptor CCR2 in WT and GILZ KO mice, and evaluated the number of infiltrating leukocytes in the livers following CCl_4_ treatment. The pharmacological downregulation of CCR2 expression was performed in vivo using siRNA embedded in LNPs^60^. This system provides an efficient and clinically approved solution for gene silencing in vivo. The common issue with siRNA is miRNA-like activity that influence on the expression of partially complementary mRNA^61^. To avoid the contribution of off-target effects on the results, we selected several siRNA sequences targeting different CCR2 mRNA sites, introduced 2′-O-methyl pyrimidines and phosphorothioates to increase specificity and decrease immune response, and used the control siRNA with the same pattern of chemical modifications and same LNPs. The high efficacy of siRNA also decreases the possibility of off-target effects. Such setup provides a solid solution for proof-of-concept studies in the liver in vivo^52,62^. In order to achieve silencing of CCR2 mRNA, we compared previously published CCR2-targeting siRNA sequence with the best scored six siRNA in RAW264.7 cells (Supplementary Table 1). The most potent siRNAs (“siCCR2”) was used in further in vivo studies. Experimental scheme for the in vivo CCR2 silencing and CCl_4_ treatment experiment is shown in Fig. S9A. Biweekly in vivo administration of LNP siCCR2 siRNA (0.5 mg/kg) led to a significant reduction in the levels of expression of *Ccr2* mRNA (Fig. S9B). WT and GILZ KO mice were pretreated with CCR2 siRNA or Luciferase siRNA (siLUC, control) and then treated with CCl_4_ or oil. Evaluation of leukocyte number in the livers at 72 h after CCl_4_ treatment revealed that the downregulation of *Ccr2* in a lack of significant differences in the leukocyte number observed in WT and GILZ KO mice (Fig. 6A). Importantly, this also led to a diminished HSC activation as evidenced by immunostaining for α-SMA (Fig. 6B), suggesting that diminishing leukocytes recruitment also dampens the enhanced pro-fibrotic HSC activation observed in GILZ KO mice. Altogether these data demonstrate that the mechanisms underlying the enhanced recruitment of leukocytes in the livers of GILZ KO mice involve CCR2–CCL2 pathway.

### GILZ is downregulated in human patients with liver fibrosis

**Fig. 7:**
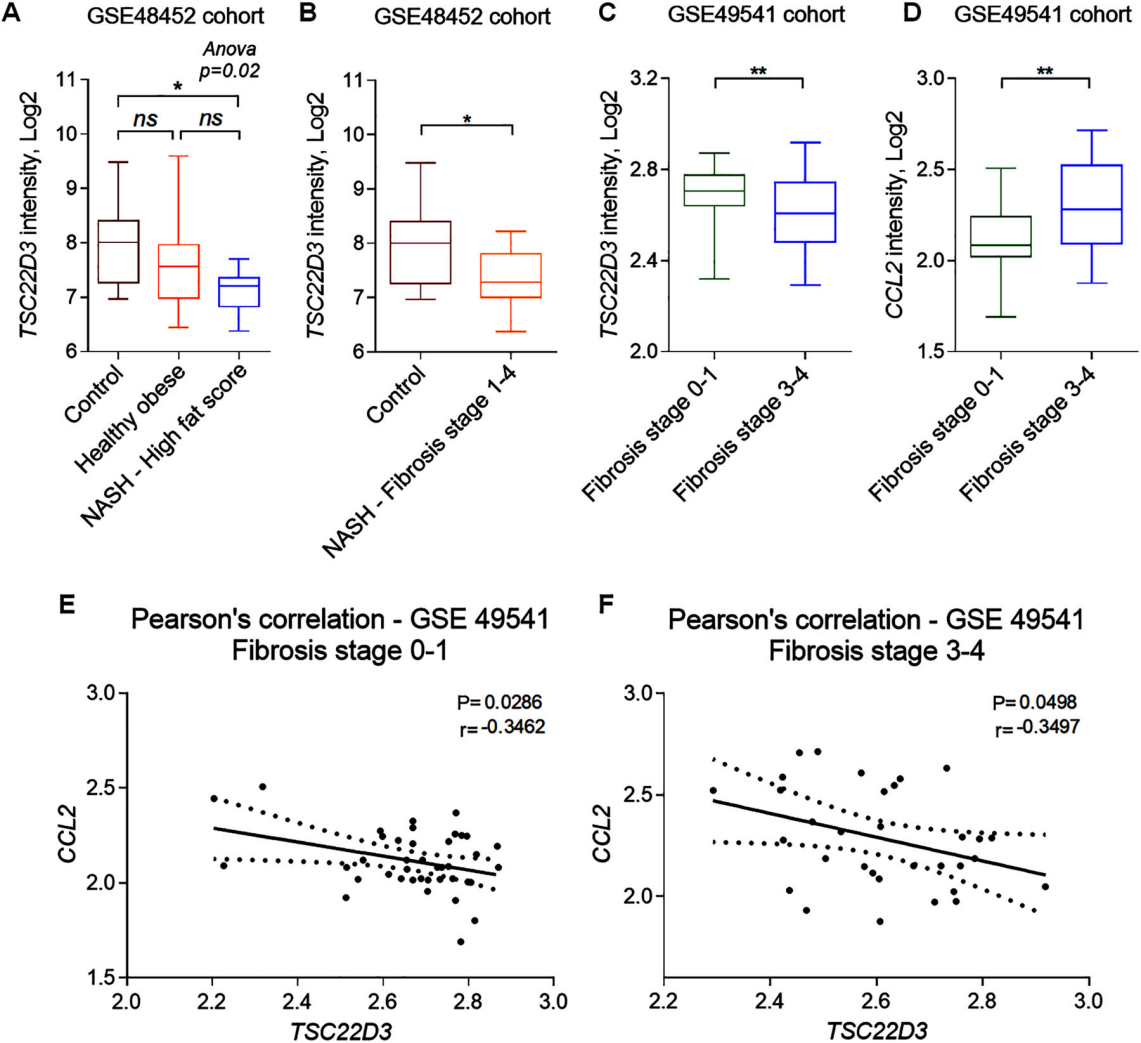
GILZ is downregulated in patients with LF. *TSC22D3* and *CCL2* mRNA expression was analyzed in liver samples from NAFLD patients, according to GSE48452 (NASH patients) and GSE49541 (NAFLD patients) cohorts accessed in Gene Expression Omnibus. **A**, **B** *TSC22D3* mRNA expression in NASH patients. Samples were classified as **A** high fat score when presenting a score of ≥70 (*n* = 9) and **B** fibrosis stages 1–4, representing the samples in this score range (*n* = 14). **C**, **D** *TSC22D3* (**C**) and *CCL2* (**D**) gene expression in NAFLD patients with mild (stages 0–1; *n* = 40) and advanced (stages 3–4; *n* = 34) fibrosis. **E**, **F** Pearson correlation between *TSC22D3* and *CCL2* mRNA expression in NAFLD patients. **E** Pearson correlation coefficient in NAFLD patients with mild (stages 0–1) and (**F**) advanced (stages 3–4) fibrosis. Data are presented as a box and whiskers plot. *p < 0.05, **p < 0.01.

In order to address the clinical relevance of GILZ–CCL2 axis in human LF, we analyzed GILZ mRNA expression in RNAseq data obtained from NASH and NAFLD patient liver samples^63,64^. We found that the mRNA expression of *TSC22D3* gene encoding for GILZ in humans was significantly lower in NASH patients with a fat score ≥70 compared to the healthy control and healthy obese patients group (Fig. 7A). The *TSC22D3* mRNA expression was also significantly lower in NASH patients presenting LF (stages 1–4), as compared to those without fibrosis (Fig. 7B). Interestingly, in an independent cohort of NAFLD patients^58^, the *tsc22d3* mRNA expression inversely correlated with the degree of LF, as it was significantly lower in patients with advanced fibrosis (stages 3–4) compared to patients presenting fibrosis stages 0–1 (Fig. 7C; GSE49451). Conversely to decreased *TSC22D3* mRNA levels, the mRNA expression of *CCL2* was significantly higher in livers of NAFLD patients with higher fibrosis score compared to those with lower fibrosis score (Fig. 7D). Pearson correlation test confirmed that the mRNA expression levels of *TSC22D3* and of *CCL2* were inversely correlated, as evidenced by a significant negative correlation scores both in samples with lower (Fig. 7E) and higher (Fig. 7F) fibrosis stages. Altogether, these data demonstrate that GILZ expression is lower in livers of human NASH and NAFLD patients presenting fibrosis compared to healthy controls, and that its expression inversely correlates with the expression of CCL2.

## Discussion

LF is associated with infectious, autoimmune, alcoholic, and metabolic liver diseases^65^. Current LF therapies include strategies based on inhibition of collagen synthesis, HSC-targeted therapies, and cytokine-blocking strategies. None of anti-fibrotic treatments have succeeded so far^66^. Thus, finding novel molecular players involved in LF can provide an important information for future drug development. GC have been applied to the clinical treatment of liver failure, alcoholic, and autoimmune hepatitis for many years^15–18,67^. GC’s contradictory results in the treatment of LF are possibly linked to their opposing effects on immune cells and HSC^20^. Chronic use of GC is accompanied by important side effects^13,68^. GC have been implicated in the pathogenesis of NAFLD due their metabolic effects on liver and fat tissues^69^. Therefore, new drugs are needed to replace GC in treatment of inflammatory diseases, including LF.

The function of GILZ in LF development has not been directly addressed yet. Using mouse model of genetic ablation of GILZ in all tissues we observed an increased CCl_4_-induced LF. The expression of cell death-related genes was not altered in GILZ KO mice, thus we concluded that the enhanced susceptibility to CCl_4_-induced LF depends on other cellular processes. LF development is strictly associated with inflammation^4,70–72^. We found that GILZ controls the leukocyte recruitment to the liver during initial and chronic phases of LF development, following CCl_4_-induced liver damage. This is consistent with the role of GILZ in regulation of leukocyte trafficking in different experimental mouse models, such as rheumatoid arthritis^35,73^, spinal cord injury^74^, and inflammatory bowel diseases^44,75^. The inflammatory reaction of the Th2 type mediated by the CD4^+^ T lymphocytes and macrophages expressing Th2 cytokines represents an important factor involved in the higher susceptibility to fibrosis^6,76–79^. Interestingly, we found that the deletion of GILZ is associated with an increased presence of CD4^+^ T cells and macrophages in the livers of CCl_4_-treated mice. Moreover, the absence of GILZ was associated with increased IL-4 and a decreased IFN-γ expression. These results suggest that GILZ deletion alters the Th1/Th2 balance during the CCl_4_-mediated inflammatory reaction in the livers, resulting in a predominant pro-fibrotic Th2 immune state. This finding is in contrast with previous finding of GILZ promoting the Th2 differentiation^31^, but is consistent with the fact that the lack of GILZ causes the Th2 shift during spinal cord injury^74^. These data demonstrate that GILZ regulates the type of the inflammatory reaction in the liver and suggest that the regulation of Th1/Th2 differentiation by GILZ might be tissue context-dependent.

Chemokine expression by resident liver cells promotes infiltration of monocytes/macrophages, NK cells, NKt cells, neutrophils, B cells, and T cells^59,79,80^. GILZ was previously shown to control the expression of CCL5 and CXCL12 chemokines^27,34^. Here, we found that CCl_4_-treated GILZ KO mice expressed higher levels of *Ccl2*. To the contrary, the expression of CCL5 and CXCL12 did not differ between the livers of WT and GILZ KO mice, suggesting that the regulation of chemokine expression by GILZ is context-dependent. Interestingly, in GILZ KO mice, we found significant increase in the recruitment of the cell populations whose trafficking is regulated by CCL2, namely, macrophages, CD4^+^ T cell, and in part, NK cells^79,81^. At the same time recruitment of granulocytes, NKt and CD8^+^ T cells did not significantly differ between WT and GILZ KO mice. Taken together these data demonstrates that the deletion of GILZ is associated with elevated CCL2 expression and the enhanced liver recruitment of CCL2-sensitive immune cells.

To demonstrate that enhanced leukocyte recruitment to liver in GILZ KO mice is dependent on CCL2 levels, we performed in vivo RNAi silencing of CCR2 receptor in the liver via in vivo administration of CCR2 siRNA in LNPs. This system was successfully employed to downregulate mRNA expression in vivo in mice and nonhuman primates^60^, and similar siRNA-LNP were approved by Food and Drug Administration (FDA) and European Medicines Agency (EMA) for the treatment of human Hereditary Transthyretin Amyloidosis^82,83^. RNA therapeutics evolved a lot during last years—a number of oligonucleotide drugs were approved by FDA and EMA and many of them are at late stages of clinical trials. Importantly, our data reveal that pharmacological downregulation of CCR2 attenuated leukocytes recruitment into the liver upon CCl_4_ challenge, thus providing a preclinical evidence of the possible therapeutic venue for the modulation of LF.

Importantly, our findings were supported by clinical data. Using public gene expression datasets, we found that the *TSC22D3* mRNA expression was lower in specific subgroups of human NAFLD patients presenting elevated LF scores^63,64^. Moreover, the expression of GILZ inversely correlated with that of CCL2 in human NAFLD patients, in line with the evidence we obtained in mice. These data suggest that GILZ–CCL2 axis may have clinical relevance for the development of human LF, and thus represents perspective therapeutic target. In conclusion, our findings highlight an important role of GILZ in the regulation of LF development and provide the basis for the development of novel therapeutics to treat inflammatory liver diseases.

## Supplementary information

Supplementary material
