# Supplementary material for: Glucocorticoid-induced leucine zipper regulates liver fibrosis by suppressing CCL2-mediated leukocyte recruitment
Source: Cell Death Dis. 2021 Apr 29;12(5):421. doi: 10.1038/s41419-021-03704-w (PMC8085011; doi:10.1038/s41419-021-03704-w)
Supplement: Supplementary file 1 — Supplementary material [file 41419_2021_3704_MOESM1_ESM.docx]

**Supplementary material**

**Glucocorticoid-induced leucine zipper (GILZ) regulates liver fibrosis by suppressing CCL2-mediated leukocyte recruitment**

by Sara Flamini et al.

Includes:

Supplementary Figures 1-9;

Supplementary Methods;

Supplementary Tables 1-2;

Supplementary References.


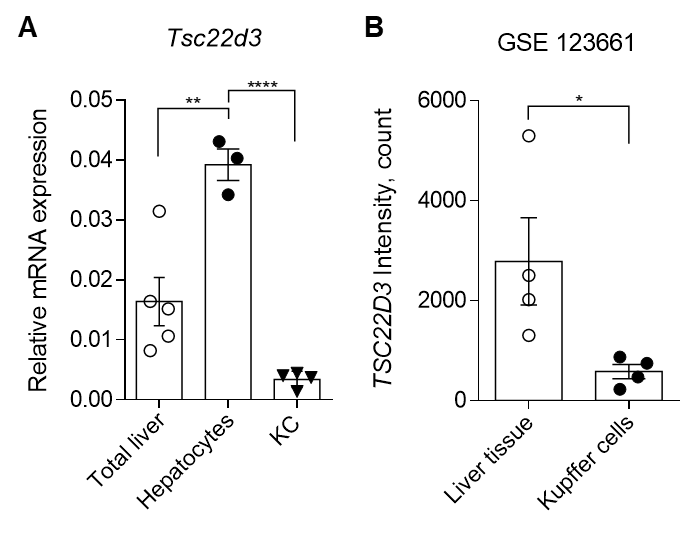


**Supplementary Figure 1. Analysis of the GILZ expression in liver cells.**

(A) qPCR analysis of mRNA expression of *Tsc22d3* total liver, hepatocytes and Kupffer cells isolated from WT mice. (B) Analysis of *TSC22D3* mRNA expression in total human liver samples and purified Kupffer cells according to GSE123661 (healthy liver tissue and isolated Kupffer cells) cohort accessed in Gene Expression Omnibus^1^. Results are presented as the means ± SEM. **<0.01, ****<0.0001, Student’s *t test*.


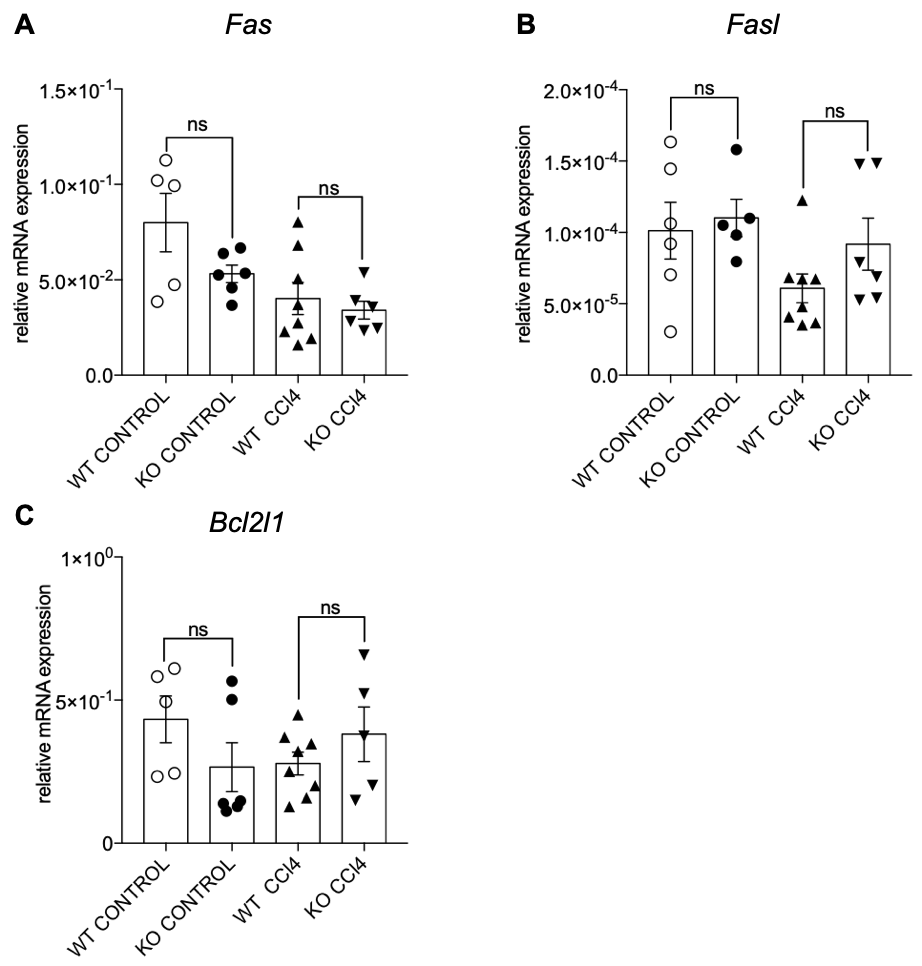


**Supplementary Figure 2. GILZ deletion does not significantly affect the expression of apoptosis related genes.**

(A-C) qPCR analysis of mRNA expression of *Fas* (A), *Fasl* (B), *Bcl2l1* (C) in the livers isolated from 4-mo-old WT and GILZ KO mice treated for 72 hours with oil (control) or CCl4. All data are presented relative to the expression of *Actb* mRNA. Each dot represents an individual mouse, bars indicate the mean. Results are presented as the means ± SEM. Data were pooled from two independent experiments (ns = not significant).


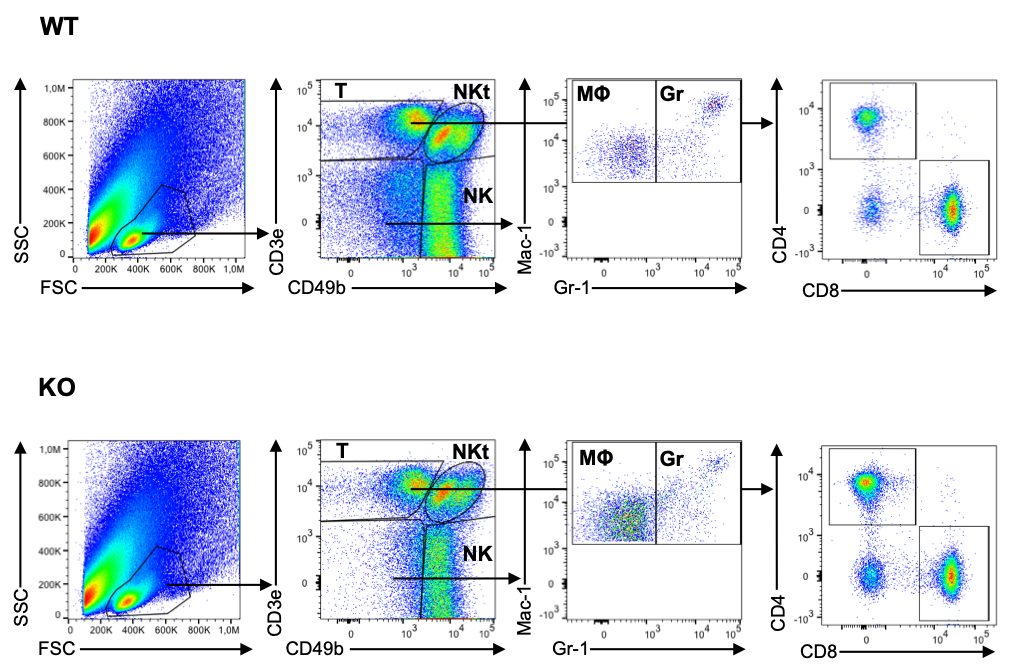


**Supplementary Figure 3. Scheme of the flow cytometry analysis of liver-infiltrating leukocyte subpopulations.**

Representative dot plots of flow cytometry analysis of leukocytes isolated from livers of WT (upper panels) and GILZ KO (lower panels) mice following CCl4-induced liver damage. Sequential gating strategy is indicated by arrows to define NK, natural killer cells; T, T cells; Gr, granulocytes; MΦ, monocytes and macrophages.


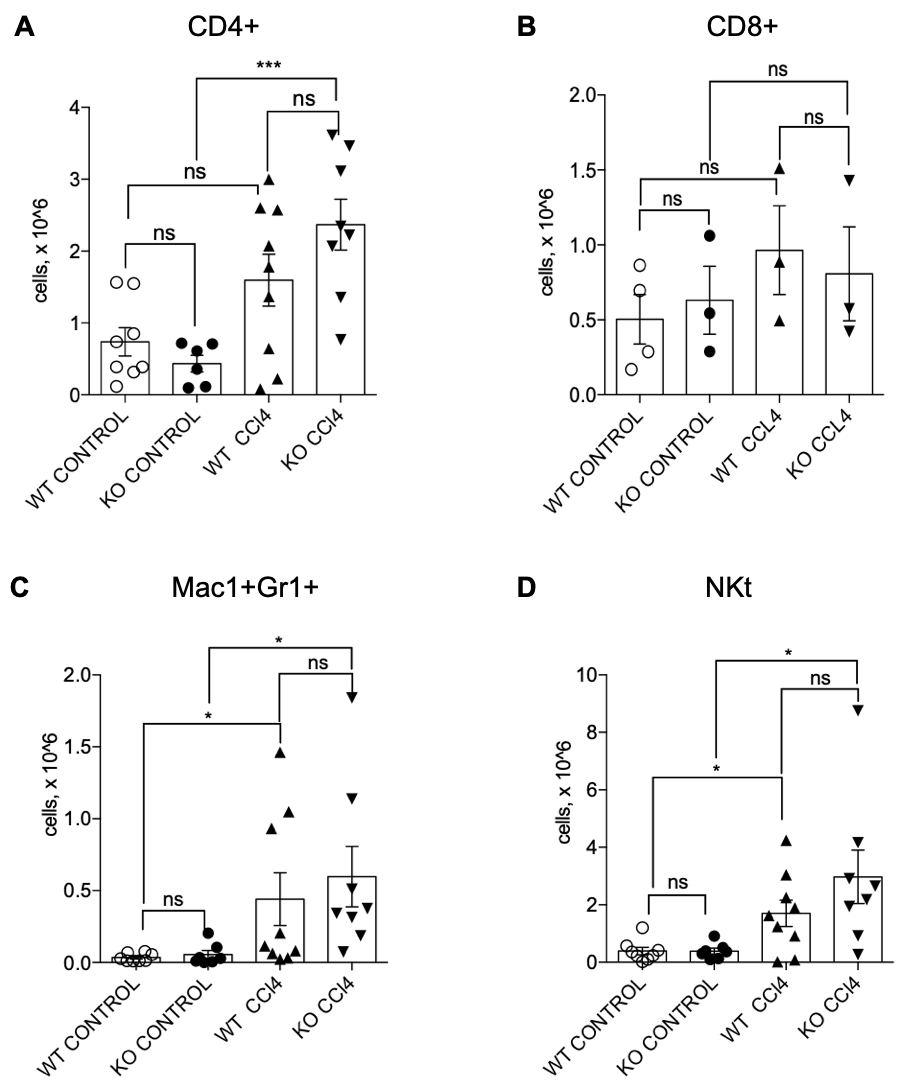


**Supplementary Figure 4. GILZ deletion does not significantly affect the recruitment of CD8+ T cells, granulocytes and NKt cells to the liver following CCl4-induced liver damage.**

(A-D) Number of CD3+ (A), CD8+ (B), Mac1+Gr1+ (C) and NKt (D) cells found in livers of 4-mo-old WT and GILZ KO mice treated with oil (control) or CCl4 for 72 hours. Data were pooled from three (A-D) or two (B-C) independent experiments. Results are presented as the means ±SEM; each dot represents an individual mouse, bars indicate the mean. (*p < 0.05,***p < 0.001, ns = not significant).


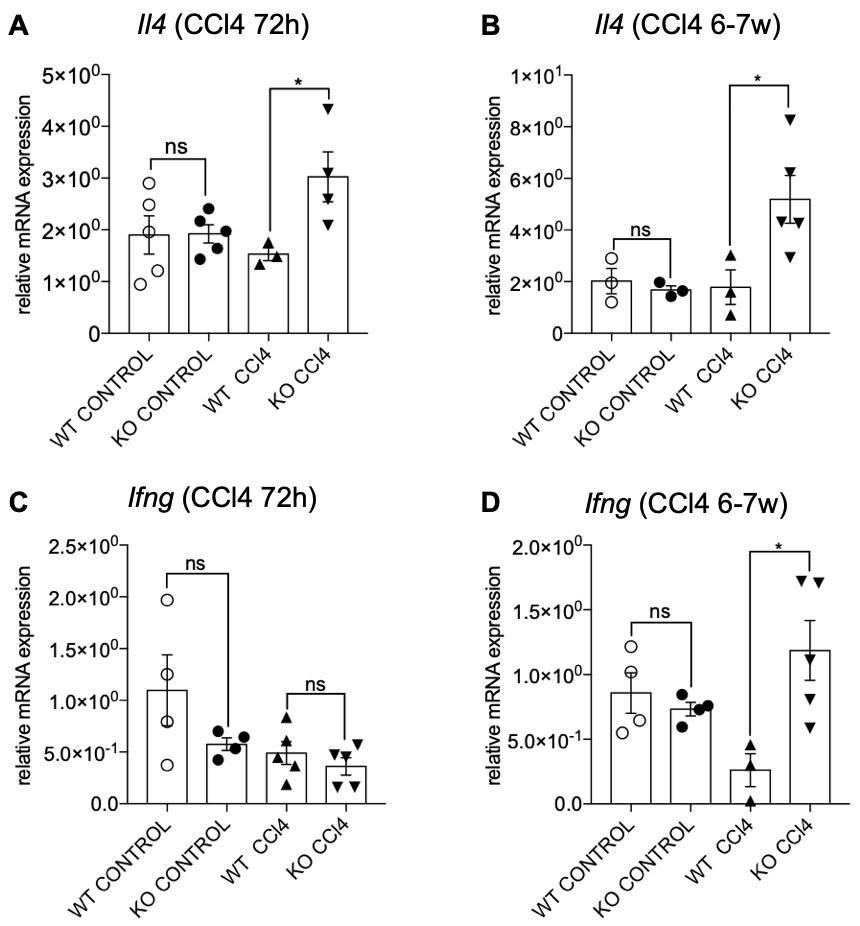


**Supplementary Figure 5. GILZ deletion results in elevated expression of *Il4* mRNA upon CCl4 treatment.**

(A-B) qPCR analysis of mRNA expression of *Il4* in livers isolated from 4-mo-old WT and GILZ KO mice treated for 72 hrs (A) or 6-7 weeks (B) with oil (control) or CCl4. (C-D) qPCR analysis of mRNA expression of *Ifng* in livers isolated from 4-mo-old WT and GILZ KO mice treated for 72 hrs (C) or for 6-7 weeks (D) with oil (control) or CCl4. All data are presented relative to the expression of *Actb* mRNA. Each dot represents an individual mouse, bars indicate the mean. Results are presented as the means ± SEM. Data were from two independent experiments (*p < 0.05, ns = not significant).


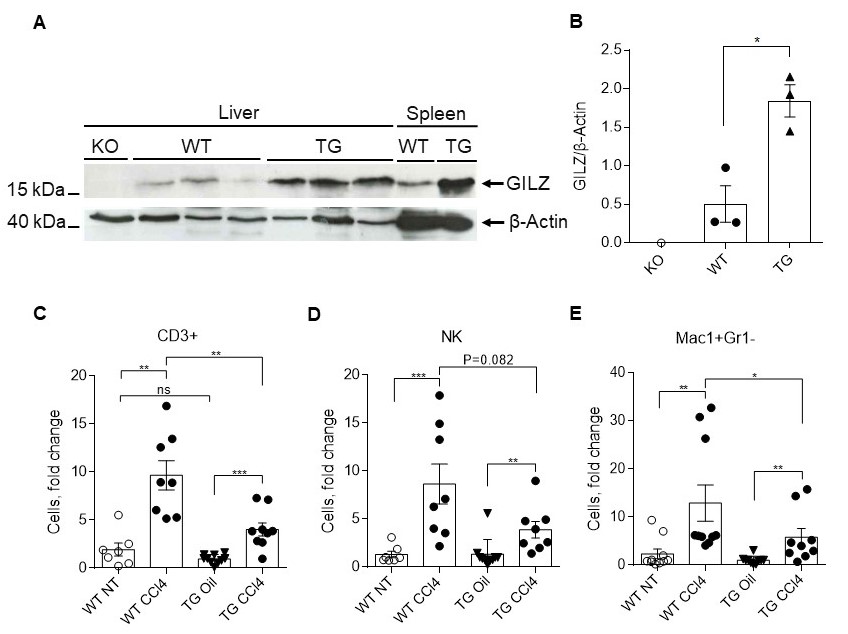


**Supplementary Figure 6. Elevated GILZ expression decreases leukocytes infiltration in murine liver.** (A) Western blot analysis of GILZ protein expression in liver and spleen of WT, GILZ KO and GILZ TG mice. (B) Densitometry analysis of GILZ protein expression relative to the expression of β-Actin. Data are shown as mean ± SEM. * P<0,05, Student’s *t test*. (C-E) Fold change in cell number of CD3+, NK cells, and macrophages in WT and TG mice in acute model of liver fibrosis. Each dot represents an individual mouse, bars indicate the mean. Results are presented as the means ± SEM. Data were from three independent experiments. *=P<0.05, **=P<0.005, ***=P< 0.001, Mann-Whitney test.


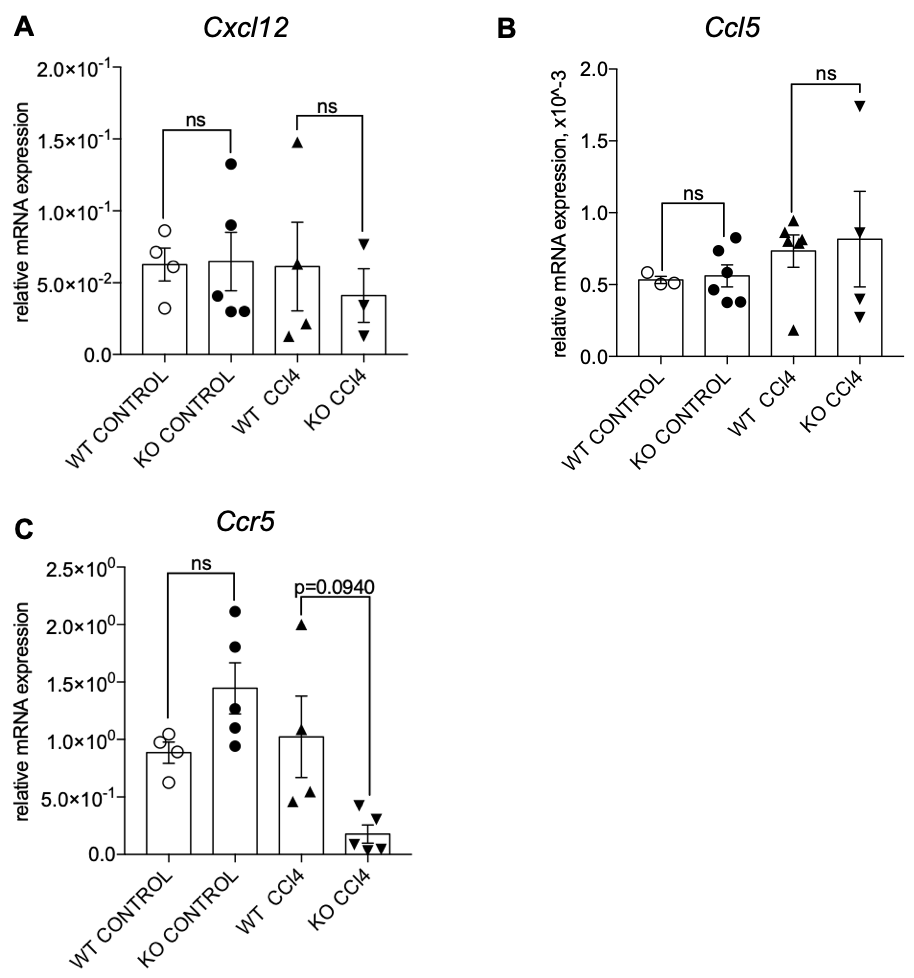


**Supplementary Figure 7. GILZ deletion does not significantly affect the expression of *Ccl5/Ccr5* and *Cxcl12.***

(A-C) qPCR analysis of mRNA expression of *Cxcl12* (A), *Ccl5* (B), *Ccr5* (C) in livers isolated from 4-mo-old WT and GILZ KO mice treated for 72 hours with oil (control) or CCl4. All data are presented relative to the expression of *Actb* mRNA. Each dot represents an individual mouse, bars indicate the mean. Results are presented as the means ± SEM. Data were pooled from two independent experiments (ns = not significant).


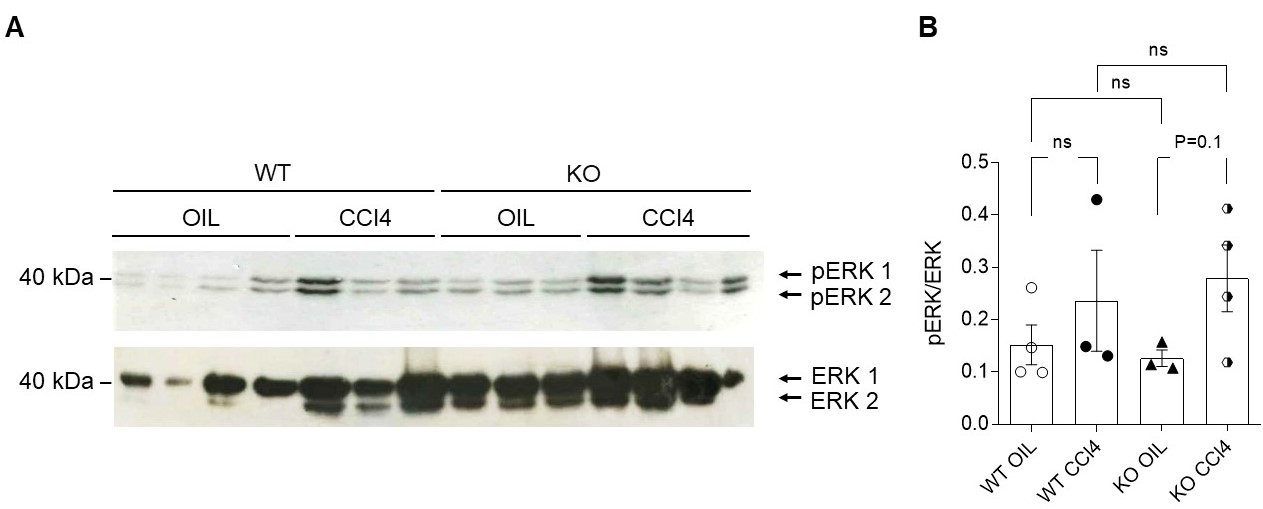


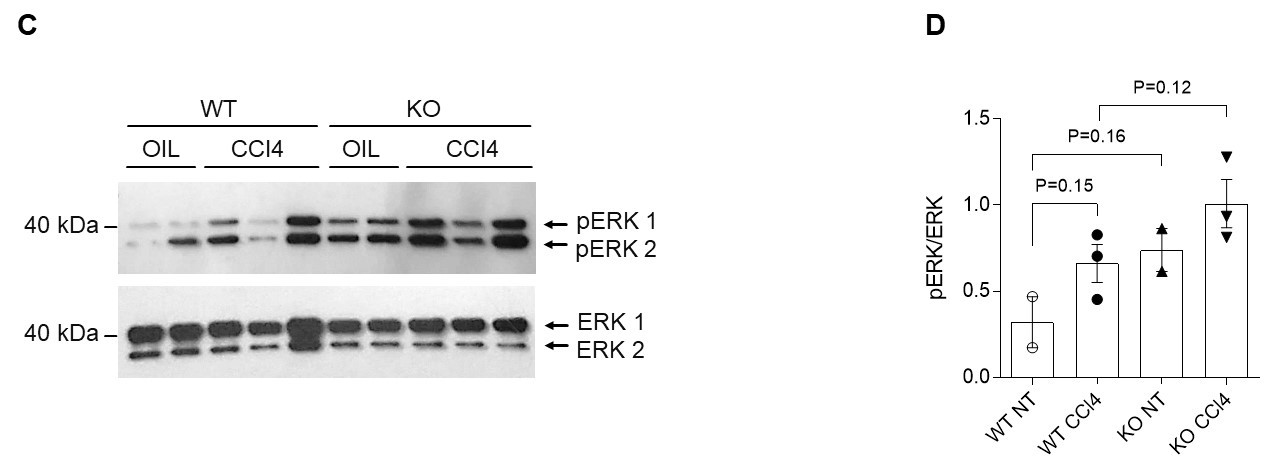


**Supplementary Figure 8. Evaluation of ERK phosphorylation in livers of WT and GILZ KO mice.**

Western blot analysis of phosphorylated ERK (upper panel) and total ERK (middle panel) in extracts of livers isolated from 4-mo-old WT and GILZ KO mice treated for 72 hours (A) or 6-7 weeks (C) with oil or CCl4. Densitometry analysis of phosphorylated ERK relative to total ERK in WT and GILZ KO mice treated for 72 hours (B) or 6-7 weeks (D) with oil or CCl4. Each dot represents an individual mouse, bars indicate the mean. Data are shown as means ± SEM. ns=not significant, Unpaired *t* test.


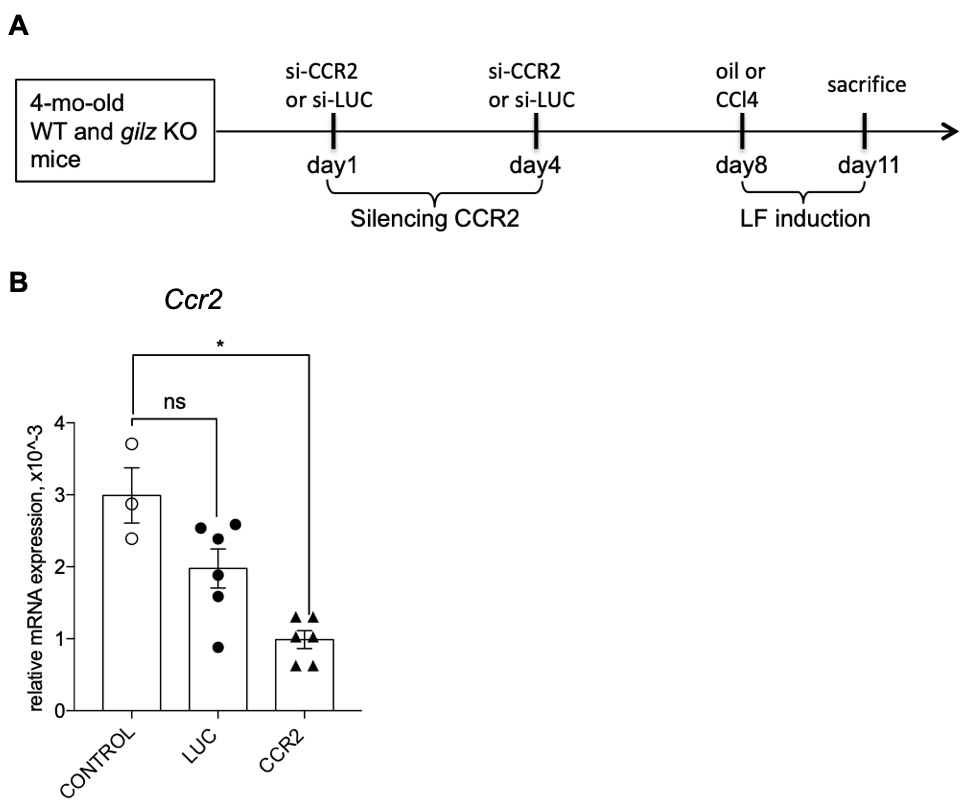


**Supplementary Figure 9. Small interfering RNA (siRNA) CCR2 *in vivo* delivery decreased *Ccr2* expression.**

(A) Experimental scheme of in vivo CCR2 silencing experiment. 4-mo-old WT and GILZ KO mice were pre-treated bi-weekly with LUC siRNA (siLUC) or CCR2 siRNA (siCCR2) and then were subsequently treated with oil (control) or CCl4 and sacrificed 72 hours following CCl4 treatment. (B) qPCR analysis of mRNA expression of *Ccr2* in 4-mo-old WT mice, untreated (control) or pre-treated with LUC (siLUC) or CCR2 (siCCR2) siRNA. Data are presented relative to the expression of *Actb* mRNA. Each dot represents an individual mouse, bars indicate the mean. Results are presented as the means ± SEM. Data were from two independent experiments (*p < 0.05, ns = not significant).

**Supplementary Methods**

**Supplementary methods for CCR2 silencing experiment.**

**Supplementary Table 1. CCR2 siRNA used in the study**

| CCR2  (PMID: 21983520) | CCR2_s | uGcuAAAcGucucuGcAAATsT |
| --- | --- | --- |
|  | CCR2_as | UUUGcAGAGACGUUuAGcATsT |
| CCR2-7 | CCR2-7s | GcAAcAuGuuGGucAuuAuTsT |
|  | CCR2-7as | AuAAUGACcAAcAUGUUGCTsT |
| CCR2-8 | CCR2-8s | GuAcuuGGcuAuuGuucAuTsT |
|  | CCR2-8as | AUGAAcAAuAGCcAAGuACTsT |
| CCR2-9 | CCR2-9s | AccuGuGGcccuuAuuuuATsT |
|  | CCR2-9as | uAAAAuAAGGGCcAcAGGUTsT |
| CCR2-10 | CCR2-10s | GAAGGuAucucuccAuAuuTsT |
|  | CCR2-10as | AAuAUGGAGAGAuACCUUCTsT |
| CCR2-11 | CCR2-11s | uuAcAcGAAGuAuccAAGATsT |
|  | CCR2-11as | UCUUGGAuACUUCGUGuAATsT |
| CCR2-12 | CCR2-12s | AuccAcGGcAuAcuAucAATsT |
|  | CCR2-12as | UUGAuAGuAUGCCGUGGAUTsT |

Uppercase – ribonucleotides, T – thymidine, lower case – 2’-O-methyl nucleotides, s – phosphorothioate

**Cell culture and transfections to test CCR2 knockdown efficiency by various siRNAs**

Mouse RAW264.7 cell line (ATCC, Manassas, VA) was grown in DMEM supplemented with 10% FBS (Gibco, Waltham, MA) and 2 mM L-glutamine (Gibco) without antibiotics. Cells were split when they reached 80% confluency. siRNA were transfected using Lipofectamine RNAiMAX (Invitrogen) according to the manufacturer’s instructions.

**cDNA synthesis and qPCR**

Total RNA was isolated using TRIzol (Thermo Fisher Scientific) according to the manufacturer's instructions. ~0.5 μg of total RNA was further treated with DNase I (Thermo Fisher Scientific), supplied with RiboLock RNase Inhibitor. For RT-qPCR, total RNA was used to synthesize cDNA using Maxima First Strand cDNA Synthesis Kit (Thermo Fisher Scientific), followed by qPCR using Maxima SYBR Green qPCR Master Mix (ThermoFisher Scientific). Conventional PCR was performed using the primers listed below.

*Ccr2* forward: GAATTGAACTTGAATCATCTGC

*Ccr2* reverse: GTCATACGGTGTGGTGGCCC

*Gapdh* forward: TGCACCACCAACTGCTTAGC

*Gapdh* reverse: GGCATGGACTGTGGTCATGAG

**Supplementary Methods Figure 1.** Efficacy of distinct siRNA for downregulaton of CCR2 mRNA expression in RAW264.7 cell line.


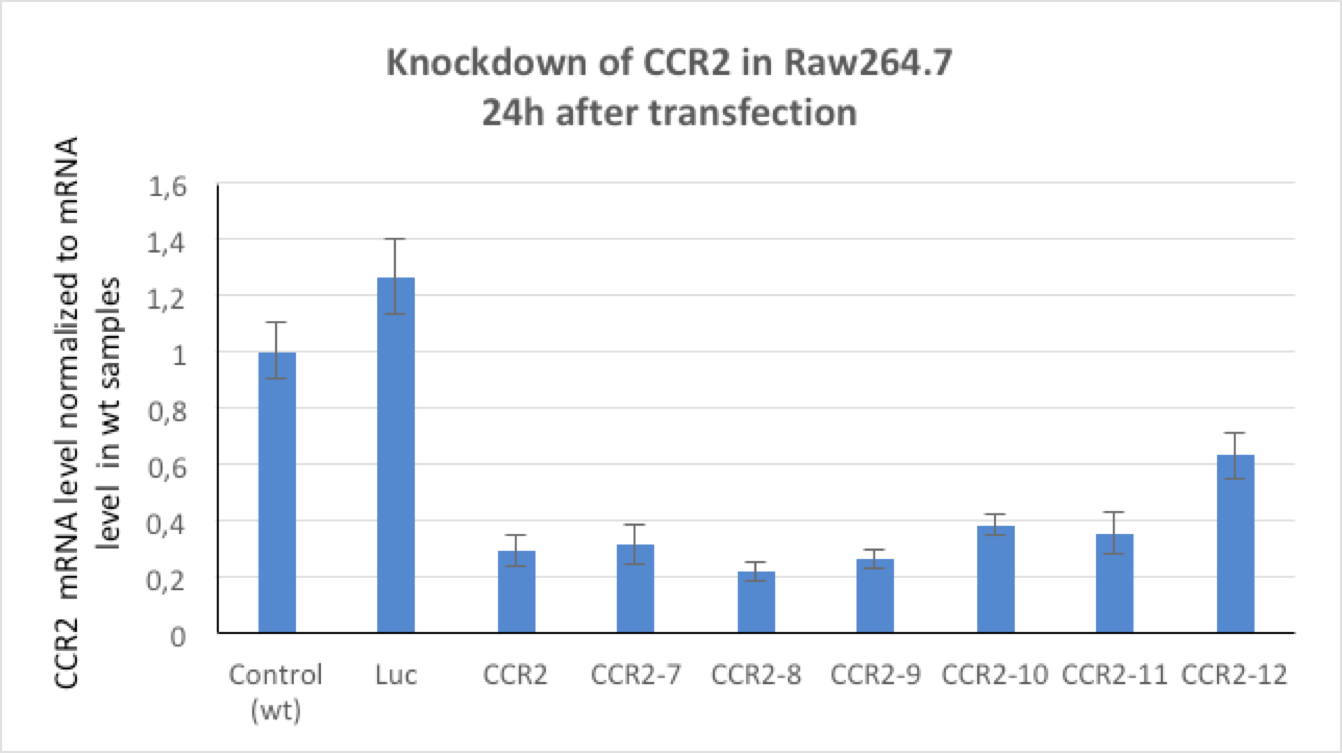


**siRNA selected for in vivo administration:**

| siRNA name | Ionizable lipid | Particle size, nm | PdI | siRNA concentration, ng/uL |
| --- | --- | --- | --- | --- |
| siCCR2 | C12-200 | 79.8 ± 2.5 | 48 | 180.5 ± 4.9 |
| siCCR2-8 | C12-200 | 94.2± 1.4 | 114 | 167.4 ± 3.2 |
| siLuc | C12-200 | 76.2 ± 1.66 | 65 | 167.7 ± 6.7 |

**Supplementary Table 2. qPCR primers using in evaluation of gene expression**

| **Gene** | **Forward** | **Reverse** |
| --- | --- | --- |
| *Actb* | CCAACCGTGAAAAGATGACC | CGTGAGGGAGAGCATAGCC |
| *Ccl2* | AAGAGGATCACCAGCAGCAG | TCTGGACCCATTCCTTCTTG |
| *Ccr2* | TGCCATCATAAAGGAGCCATACCTG | TGTGGTGAATCCAATGCCCTCT |
| *Ccl5* | CTTGGGGATGCCACTCAGTA | AGATGCCCATTTTCCCAGGA |
| *Ccr5* | TGCAGTCCTCATTTTCCACA | CCTACAGCGAAACAGGGTGT |
| *Ifng* | GCTTTGCAGCTCTTCCTCAT | ATCCTTTTGCCAGT |
| *Asma* | GTCCCAGACATCAGGGAGTAA | TCGGATACTTCAGCGTCAGGA |
| *Il4* | CCTCACAGCAACGAAGAACA | ATCGAAAAGCCCGAAAGAGT |
| *Cxcl12* | CAACGTCAAGCATCTGAAAATC | GATCCACTTTAATTTCGGGTCA |

**Supplementary References:**

1. Pose, E., Coll, M., Lozano, J.J., Ginè, P. Transcriptomic analysis of Kupffer cells in patients with cirrhosis. Dataset GSE123661 in GEO Omnibus (<https://www.ncbi.nlm.nih.gov/geo/query/acc.cgi?acc=GSE123661>). Accessed on 22/02/2021.
